# Supplementary material for: Let’s Agree to Disagree on Operative versus Nonoperative (LADON) treatment for proximal humerus fractures: Study protocol for an international multicenter prospective cohort study
Source: PLoS One. 2022 Feb 25;17(2):e0264477. doi: 10.1371/journal.pone.0264477 (PMC8880817; doi:10.1371/journal.pone.0264477)
Supplement: S1 File — (DOCX) [file pone.0264477.s002.docx]

**Handling and storage of data and documents**

Data is handled confidentially. An individual subject will be connected to a subject identification code. The code is not based on the patient’s initials and birth-date but on number of participation. The handling of personal data will comply with the AVG (Algemene Verordening Gegevensbescherming). The key file will contain the subject identification codes and patient hospital file numbers. Only the PrincipaI investigator and coordinating investigator will have access to the key file and all investigators will have access to the pseudonymised data set at the local hospital. The key file will not leave the hospital and transferring data will be performed via https://www.surffilesender.nl/ only. The research data will be will be acquired using the electronic capture tool CASTOR EDC and processed and stored in the file format SPSS. To be able to reproduce the study findings and to help future users to understand and reuse the data all changes made to the raw data and all steps taken in the analysis will be documented in a syntax document. Research data will be stored on the research network disc of the division. The research data will be archived on the research network disc of the division for 15 years after the study has ended.
