# Supplementary material for: Let’s Agree to Disagree on Operative versus Nonoperative (LADON) treatment for proximal humerus fractures: Study protocol for an international multicenter prospective cohort study
Source: PLoS One. 2022 Feb 25;17(2):e0264477. doi: 10.1371/journal.pone.0264477 (PMC8880817; doi:10.1371/journal.pone.0264477)
Supplement: S2 File — (DOCX) [file pone.0264477.s003.docx]

| Data category | Information[^32^](https://www.spirit-statement.org/spirit-statement/references#32) |
| --- | --- |
| Primary registry and trial identifying number | Netherlands Trial register NL9357 |
| Date of registration in primary registry | 24-3-2021 |
| Secondary identifying numbers | N.A. |
| Source(s) of monetary or material support | DePuy Synthes |
| Primary sponsor | University medical center Utrecht |
| Secondary sponsor(s) | N.A. |
| Contact for public queries | Ruben Hoepelman, MD  rjhoepelman@gmail.com  Phone: 0616079764  UMC Utrecht |
| Contact for scientific queries | Ruben Hoepelman, MD  rjhoepelman@gmail.com  Phone: 0616079764  UMC Utrecht |
| Public title | Let's Agree to Disagree on Operative versus Nonoperative treatment for proximal humeral fractures. An international multicentre prospective cohort study |
| Scientific title | *Nonoperative versus Operative Treatment for proximal humeral fractures. An International multicentre prospective cohort study.* |
| Countries of recruitment | Netherlands, Switzerland |
| Health condition(s) or problem(s) studied | Proximal humeral fractures, Isolated greater tuberosity fractures |
| Intervention(s) | Active comparator:*Operative treatment, consisting of open reduction and internal plate fixation (ORIF), minimal invasive plate osteosynthesis (MIPO), intramedullary nailing, or reverse shoulder arthroplasty*  Nonoperative treatment |
| Key inclusion and exclusion criteria | Inclusion criteria  All patients (>18 years) presenting acute displaced proximal humeral fracture involving minimally the surgical neck, including isolated greater tuberosity fractures will be eligible for inclusion.  Exclusion criteria  - Open fracture - Pre-existing co-morbidities which preclude operative treatment - Pathological fractures, associated dislocation of injured shoulder joint - Associated ipsilateral upper extremity fractures - Concomitant soft tissue injury or neurovascular injuries requiring operative treatment - Delayed presentation (> 3weeks after injury) - Treatment for re-fractures - Cognitive impairment - Non-Dutch, non-German or non-English speaking patients - Patients not resident in the hospitals area and unavailable for follow-up. |
| Study type | Study type: Observational  Control group: Active  Grouping: Parallel  Arms: 2, non-randomized  Masking: None |
| Date of first enrolment | 1 Juy 2020 |
| Target sample size | 220 |
| Recruitment status | Recruiting |
| Primary outcome(s) | QuickDash score at 12 months |
| Key secondary outcomes | - The subjective shoulder value (SSV) - EuroQol five dimensional questionnaire (EQ5D) - Numerical Rating Scale (NRS) Pain score - Return to sports and work activity - Complications - Revision surgery - implant removal and related complications |
